# Experimental demonstration of plasmon enhanced energy transfer rate in NaYF_4_:Yb^3+^,Er^3+^ upconversion nanoparticles

**DOI:** 10.1038/srep18894

**Published:** 2016-01-07

**Authors:** Dawei Lu, Chenchen Mao, Suehyun K. Cho, Sungmo Ahn, Wounjhang Park

**Affiliations:** 1 Department of Electrical, Computer and Energy Engineering, University of Colorado, Boulder, CO 80309-0425, USA; 2 Materials Science and Engineering Program, University of Colorado, Boulder, CO 80309-0425, USA

## Abstract

Energy transfer upconversion (ETU) is known to be the most efficient frequency upconversion mechanism. Surface plasmon can further enhance the upconversion process, opening doors to many applications. However, ETU is a complex process involving competing transitions between multiple energy levels and it has been difficult to precisely determine the enhancement mechanisms. In this paper, we report a systematic study on the dynamics of the ETU process in NaYF_4_:Yb^3+^,Er^3+^ nanoparticles deposited on plasmonic nanograting structure. From the transient near-infrared photoluminescence under various excitation power densities, we observed faster energy transfer rates under stronger excitation conditions until it reached saturation where the highest internal upconversion efficiency was achieved. The experimental data were analyzed using the complete set of rate equations. The internal upconversion efficiency was found to be 56% and 36%, respectively, with and without the plasmonic nanograting. We also analyzed the transient green emission and found that it is determined by the infrared transition rate. To our knowledge, this is the first report of experimentally measured internal upconversion efficiency in plasmon enhanced upconversion material. Our work decouples the internal upconversion efficiency from the overall upconverted luminescence efficiency, allowing more targeted engineering for efficiency improvement.

The research of frequency conversion by optically active ions has a long history stretching back to the mid-1960s^1^. Generally, the ions are successively pumped multiple times *via* the long-lived intermediate energy levels from which they are excited further into the higher energy levels either by directly absorbing another photon or through energy transfer from the nearby sensitizer ions. The former is called excited state absorption (ESA) and the latter energy transfer upconversion (ETU). In either case, the ions emit photons with frequencies higher than that of the incident photon. Compared to other frequency upconversion techniques, e.g. high harmonic generation, parametric oscillation and two-photon absorption, the ETU and ESA are known to be far more efficient^1^. Thanks to the high efficiency, these processes do not require phase matching and can be excited by incoherent light source at low intensities. With the advance of nanocrystals research in recent years, high quality upconversion nanoparticles can be prepared routinely, spawning a wide range of new applications in lighting^2^, displays^3^, solar energy conversion^4^, biosensing^5^ and biomedical imaging^6^,^7^. More recently, novel applications such as security ink^8^ and photoswitching^9^ are also being explored.

For widespread applications, however, the efficiency needs to be further improved. One of the most efficient upconversion materials is NaYF_4_:Yb^3+^,Er^3+^, which is typically prepared in powder form and exhibits upconversion efficiencies up to 4% at around 10 W/cm^2^ excitation intensity^10^. For some applications such as solar cell and biomedical imaging, nanometer size upconversion particles are required. With the reduced size, the overall luminescence efficiency decreases dramatically due to the more severe quenching to surface defects^11^. Plasmonics offer a promising avenue to overcome this problem and achieve high efficiency. By placing the plasmon resonance at the excitation frequency, both the absorption and energy transfer processes can be enhanced^12^,^13^. The enhancement of upconversion photoluminescence (PL) can be several hundredfold^14^,^15^. Surface plasmon can also enhance the emission process, if the plasmon resonance is aligned with the emission frequency^16^, through the well-known Purcell effect^17^. There have been many reports on plasmonic enhancement of upconverted luminescence, which has been thoroughly reviewed recently^18^,^19^. Despite the rapidly increasing number of publications in this subject, the precise mechanism of plasmon enhancement for the upconversion processes is not yet fully understood^12^,^13^,^19^. Luminescence upconversion is a complex process consisting of multiple steps of absorption, Förster energy transfer and emission. While the plasmon enhancement of absorption and emission is extensively studied and well understood, the plasmon enhancement of Förster energy transfer process has been largely unknown. There has so far been no quantitative measurement of enhanced energy transfer upconversion rate, although it is critical to know whether plasmon can enhance energy transfer upconversion rate and what the potential maximum enhancement is.

In this paper, we report a systematic study on the dynamics of the upconversion processes in NaYF_4_:18%Yb^3+^,2%Er^3+^ upconversion nanoparticles (UCNPs) deposited on a plasmonic nanograting structure. By monitoring the transient near infrared (NIR) PL under different excitation power densities, we observed faster PL rise and decay with increasing excitation power density until they reached saturation. From the analysis of rate equations, we found that the faster transient NIR PL arises from the increased ETU rate. From the experimental data, we were able to determine the ETU rates and found that the ETU rate was increased by a factor of 2.7. To the best of our knowledge, this is the first report of experimentally measured ETU rate in a plasmon enhanced upconversion material. Not only does it provide a direct proof of plasmon enhancement of energy transfer process but it also gives a quantitative measure of how much enhancement is achievable. In addition, we also studied the transient green PL for different excitation conditions and found that the transient green emission is governed by the NIR transition rate. Our work elucidates the role of plasmon resonance in the ETU system, and provides a straightforward method to directly measure the energy transfer rate and internal upconversion efficiency.

## Results

### Sample description

The UCNPs used in our study are β-NaYF_4_:18%Yb^3+^/2%Er^3+^ nanoparticle synthesized by the co-precipitation method^20^,^21^. Three layers of UCNPs were deposited on top of a one-dimensional silver nanograting using the layer-by-layer method^22^,^23^,^24^. We also deposited a 30 nm Si_3_N_4_ layer between the silver nanograting and UCNP layer to alleviate quenching by metal. The silver nanograting was designed to support surface plasmon resonance at the UCNP absorption wavelength of 980 nm in the final structure. The reference sample was prepared in exactly the same way except that the silver nanograting was replaced by a flat silver film. The flat film sample makes an ideal reference for several reasons. Since the total decay rate is the sum of intrinsic decay rate and energy transfer rate, an ideal reference should exhibit the same intrinsic decay rate as the nanograting sample. This way, any observed changes in the total rise or decay rate should reflect the changes in energy transfer rate. Our NIR decay measurements showed the UCNPs on flat silver film and nanograting exhibited identical decay and thus the same intrinsic decay rate while UCNPs on a glass substrate showed significantly slower decay due to the absence of metal quenching^12^. Also, the plasmon enhanced absorption should be negligible due to poor coupling of incident light. Furthermore, our first principles calculations showed no enhancement on energy transfer rate for the flat silver film at 980 nm (see supplementary information Fig. S10). Thus, the flat silver film provides an ideal substrate for the reference sample.

The transmission electron micrograph of the UCNPs, scanning electron micrograph of the nanograting structure, and reflectance spectra of the samples have been presented in the supplementary information (SI). They showed high sample quality with strong plasmon resonance at 980 nm, as designed.

### Steady state upconverted photoluminescence

**Figure 1:**
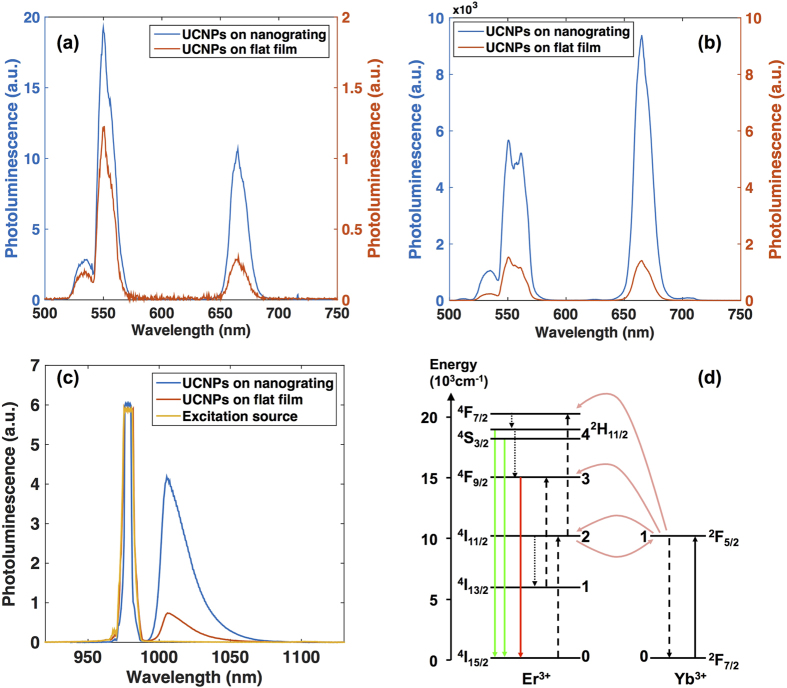
(**a,b**) are visible PL spectra of UCNPs on silver nanograting (blue, refer to left y-axis) and on flat silver film (red, refer to right y-axis) measured under 980 nm laser excitation with 1 kW/cm^2^ and 102 kW/cm^2^ power density, respectively. The photoluminescence is under arbitrary unit with the same scale. (**c**) NIR PL spectra of UCNPs on silver nanograting (blue, 5 s integration time), on silver film (red, 10 s integration time), and the 980 nm laser excitation source (yellow, 10 s integration time). The PL spectra are modified by the transmittance of a 997 nm long pass edge filter, which attenuates the scattered laser light. (**d**) Energy levels of Yb^3+^ and Er^3+^ ions relevant to the energy transfer upconversion processes. The black solid arrow indicates initial absorption of the incident photons. Dashed and dotted arrows indicate subsequent energy transfer processes and non-radiative relaxations, respectively. The green and red arrows indicate the final upconverted luminescence.

We conducted PL spectroscopy for both the UCNPs on nanograting (nanograting sample) and the UCNPs on flat silver film (reference sample) under 980 nm laser excitation with various power densities. Figure 1(a,b) shows the visible PL spectra taken at excitation power densities of 1 kW/cm^2^ (weak excitation condition) and 102 kW/cm^2^ (strong excitation condition), respectively. The green and red emission bands correspond to the ^4^S_3/2_, ^2^H_11/2_ → ^4^I_15/2_, and ^4^F_9/2_ → ^4^I_15/2_ transitions of Er^3+^ ion, respectively^10^,^20^,^25^. It is clear that the UCNPs on nanograting emit much stronger visible PL compared to the UCNPs on flat silver film. Under the weak excitation conditions, the enhancements for the green and red emission bands are 16 fold and 39 fold, respectively. In the strong excitation regime, they are reduced to 3.1x and 4.2x, respectively. A recent study showed that the surface plasmon enhances both the energy transfer upconversion and absorption in the weak excitation regime while it enhances only the absorption in the strong excitation regime^12^. Additionally, we measured the PL spectra at NIR frequencies shown in Fig. 1(c) for the nanograting sample, reference sample and 980 nm excitation laser source. The NIR emission of UCNPs originates from the ^2^F_5/2_ → ^2^F_7/2_ transition of Yb^3+^ ions and ^4^I_11/2_ → ^4^I_15/2_ transition of Er^3+^ ions. The stronger NIR emission from the nanograting sample is attributed to the surface plasmon resonance at excitation frequency, which may enhance both the absorption and emission processes.

The ETU mechanism for luminescence upconversion in Yb^3+^, Er^3+^ co-activated materials has been studied previously^26^,^27^. The major processes are shown schematically in Fig. 1(d). Due to much higher doping density and larger absorption cross-section^28^, most of the absorption is done by the donor ions, Yb^3+^. Thus, upon absorption of an incident NIR photon, an Yb^3+^ ion is pumped into the excited state ^2^F_5/2_. The excited Yb^3+^ ion can then transfer its energy to a nearby Er^3+^ *via* the Förster energy transfer process, which simultaneously de-excites the Yb^3+^ ion to the ground state, ^2^F_7/2_, and excites a nearby Er^3+^ ion into the intermediate energy level ^4^I_11/2_. If the energy transfer takes place one more time before the excited Er^3+^ ion decays back to its ground state, the Er^3+^ ion is excited to the ^4^F_7/2_ level and then quickly decays non-radiatively to the ^2^H_11/2_ and ^4^S_3/2_ levels^29^,^30^ from which the green luminescence takes place. A fraction of Er^3+^ ions in the ^4^S_3/2_ state would decay non-radiatively into the slightly lower energy level ^4^F_9/2_ where the red luminescence originates. One additional path for the red emission comes from the energy transfer upconversion of Er^3+^ ion from the ^4^I_13/2_ level which is populated by non-radiative decay from the ^4^I_11/2_ level. There are other processes that compete with upconversion process such as radiative and non-radiative decays from ^2^F_5/2_ level of Yb^3+^ ions and ^4^I_11/2_ level of Er^3+^ ions, back energy transfer from Er^3+^ ions to Yb^3+^ ions, and cross-relaxation of Er^3+^ ion pairs. The cross-relaxation of Er^3+^ ions is a major quenching mechanism at high Er^3+^ concentrations^27^,^31^. Also, there are three-photon upconversion processes, resulting in blue emission and also contributing to the green and red emission^20^. However, our PL spectra show that the three-photon processes make much smaller contributions than the two-photon processes and are therefore omitted in our analysis^25^,^32^,^33^.

### Transient NIR emission

The NIR emission is important because both the absorption and energy transfer processes take place at this frequency. The dynamics of the intermediate level, from which the NIR PL arises, is ultimately determined by the competition between the ETU rate and the decay rate. By measuring the transient NIR PL for different excitation conditions, we can explore the energy transfer processes and the influence of plasmonics on these processes. The details of transient NIR PL measurement setup are described in the Methods.

**Figure 2:**
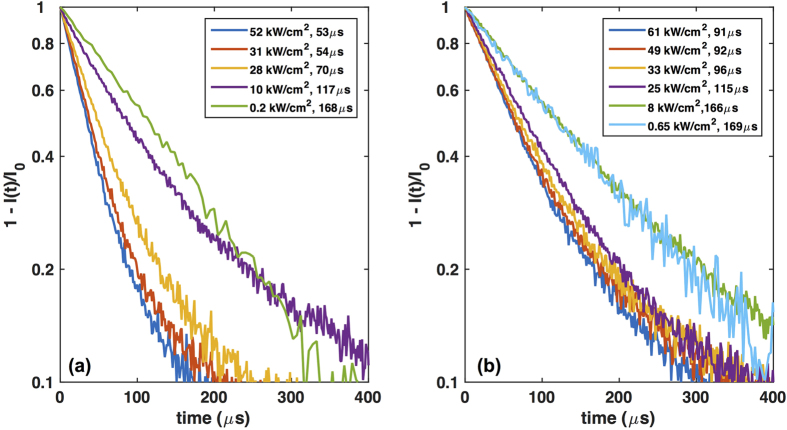
Rise of UCNPs emission at 1002 nm on silver nanograting (**a**) and on silver film (**b**). The experimentally measured rise curve is normalized to steady state PL intensity and then subtracted from unity, 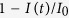. The legends describe the excitation power density and the measured rise time.

We first describe the rise of the transient NIR PL following the leading edge of the square pulse excitation. The NIR PL rise curves (Fig. 2) for both the nanograting and reference samples are fitted well by single exponential functions from which the rise rate W_D1_ can be extracted. It is noted that we normalize the transient NIR PL with the steady state PL intensity, and subtract it from unity so that we may visualize the rise process in a similar fashion to the decay process and extract the relevant rate constants. The rise time is shown in Fig. 2 legends for both nanograting and reference samples under various excitation conditions.

Under weak excitation, the NIR rise time is close to 170 μ*s* for both nanograting sample and reference sample. As the excitation power density is increased, the rise becomes significantly faster for both nanograting and reference samples. It is important to note that the rise rate of nanograting sample is always faster than that of reference sample under similar excitation conditions, indicating plasmon enhancement of energy transfer rate.

**Figure 3:**
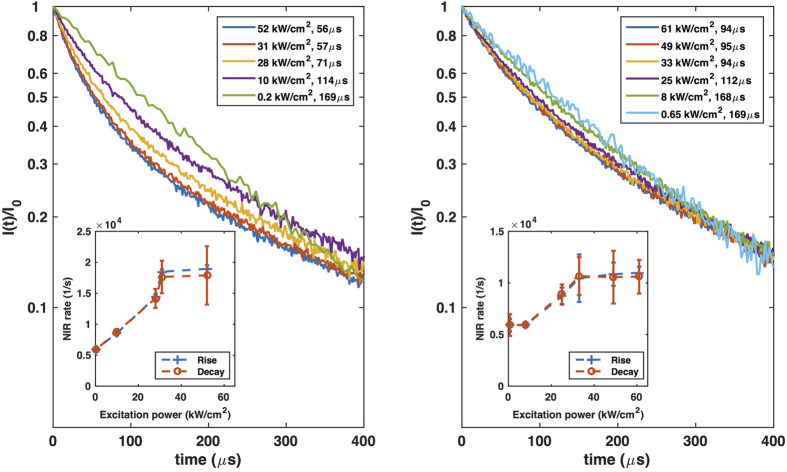
Decay of UCNPs emission at 1002 nm on silver nanograting (**a**) and on silver film (**b**). All experimental PL decay are normalized to the steady state PL intensity, and plotted in logarithmic scale. The excitation power density and decay time are listed in legends. The insets show the NIR PL rise rate and decay rate fitted from NIR PL rise and decay respectively for nanograting and reference samples under different excitation power densities. The error bar is with 95% confidence bounds.

In addition to the measurement of transient NIR PL rise process, we also measured the decay of NIR PL following the trailing edge of the square pulse excitation. The measured PL decay is normalized to the steady state PL intensity, and shown in semi-log plots in Fig. 3. Again, we observe faster decay with stronger excitation. Unlike the rise process, however, the decay starts with single exponential but deviates from it more quickly. For this reason, only the very beginning of the decay has to be fitted with an exponential function to extract the decay time, which inevitably contains larger uncertainty. They are shown in Fig. 3 legends for both nanograting and reference samples. The decay rate defined as the inverse of decay time is plotted together with the rise rate 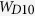 in the inset of Fig. 3. It is clear that the decay rate is always nearly equal to the rise rate under the same excitation conditions for each sample. It is slightly smaller at strong excitation due to the absence of pumping. As shown later, a more detailed analysis reveals that the initial part of rise and decay of transient NIR PL represent exactly the same physical processes with only the difference of excitation rate. However, the rise of transient NIR PL follows single exponential curve for a longer time period, as explained in more detail in the supplementary information, resulting in better fitting quality. Therefore, it is preferable to analyze the rise of transient NIR PL for the energy transfer processes study.

For complete understanding of the plasmon enhancement mechanism, we set up rate equations describing all key processes affecting the ETU mechanism. In our rate equations, the ^4^F_7/2_ level of Er^3+^ is not included as the relaxation from the ^4^F_7/2_ level to the lower-lying ^2^H_11/2_ and ^4^S_3/2_ levels is known to be extremely fast^29^,^30^,^34^. Also, the ^2^H_11/2_ and ^4^S_3/2_ levels are close enough to be considered as a single level. The complete set of rate equations is then written as,


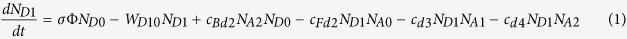



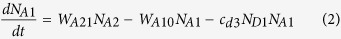



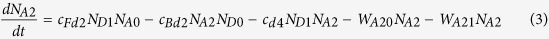



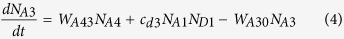



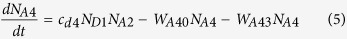



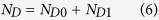



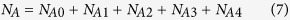


Here N_i_ is the density of ions in the energy level i. The subscripts 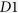 and 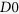 represent the ^2^F_5/2_ and ^2^F_7/2_ levels of donor ion Yb^3+^, respectively, and 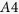, 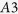, 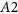, 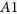, and 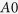 indicate the ^4^S_3/2_, ^4^F_9/2_, ^4^I_11/2_, ^4^I_13/2_ and ^4^I_15/2_ levels of acceptor ion Er^3+^, respectively. W is the decay rate from the initial state to final state denoted by the subscript. For example, 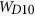 is the decay rate of donor ion from 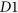 to 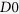 state including both radiative and non-radiative decays. 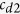, 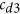 and 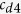 are the energy transfer coefficients for the Förster energy transfer processes between the donor and the acceptor in 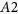, 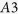 and 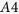 energy levels, respectively. The subscripts, F and B, indicate the forward (donor to acceptor) and backward (acceptor to donor) energy transfers. Finally, 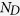 and 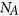 are the doping densities of donor and acceptor, respectively, 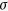 ≈ 0.24 × 10^−20^ cm^2^ is the absorption cross-section of the donor ion^35^ averaged over different polarizations at 980 nm, and 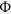 is the incident light flux. It is noted that the three-photon ETU processes are not included in the rate equations as they should in general have much lower probability than the two-photon mechanism^10^,^36^. Also omitted for simplicity are the transitions from the ^4^S_3/2_ level to the intermediate energy levels, ^4^I_11/2_ and ^4^I_13/2_, which are generally weak although they may become significant when ^4^S_3/2_ population is high. Furthermore, we consider only the backward transfer from the acceptor ^4^I_11/2_ level to donor as the lifetimes of the higher excited states of the acceptor are regarded too short to exhibit non-negligible back transfer^34^.

The NIR PL as shown in Fig. 1(c) may come from the radiative decay of both the donor (Yb^3+^) ions in 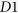 (^2^F_5/2_) level and the acceptor (Er^3+^) ions in 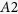 (^4^I_11/2_) level. The population of 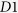 and 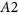 levels is described by equations (1) and (3), respectively. Combining these two rate equations we get the complete description of the transient NIR PL:


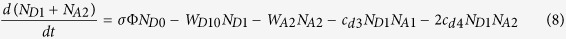


Here, 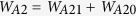 is the total decay rate of acceptor ion in level 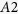. In our UCNPs, the donor doping concentration is roughly one order of magnitude larger than the acceptor concentration. It is therefore reasonable to assume that the excited donor population 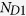 is also much larger than the excited acceptor population 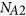. And the decay rates 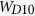 and 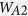 have similar value due to substantial quenching by metal^12^. Therefore, the depopulation rate 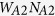 may be considered small compared to the depopulation rate 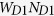. Equation (8) can then be simplified to:


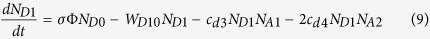


According to the experimental NIR PL rise shown in Fig. 2, the population of 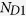 at the very beginning of the rise process can be expressed by a single exponential 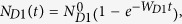 where 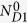 and 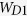 are the steady state population at 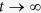 and the rise rate of 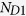, respectively. Similarly, the population of 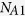 and 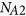 can be approximated by single exponential functions: 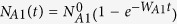 and 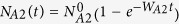. Once again, 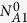 and 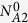 are the steady state populations, and 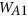 and 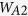 are the rise rates of 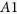 and 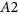 levels, respectively. The ground state population 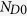 equals to the doping concentration 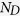 subtracted by 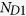. Substituting the expressions of 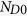, 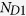, 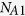, and 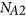 into equation (9), and solving for 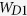 yields,


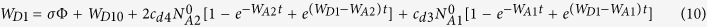


For 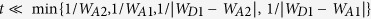, the rate 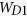 is approximated to


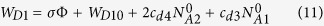


**Figure 4:**
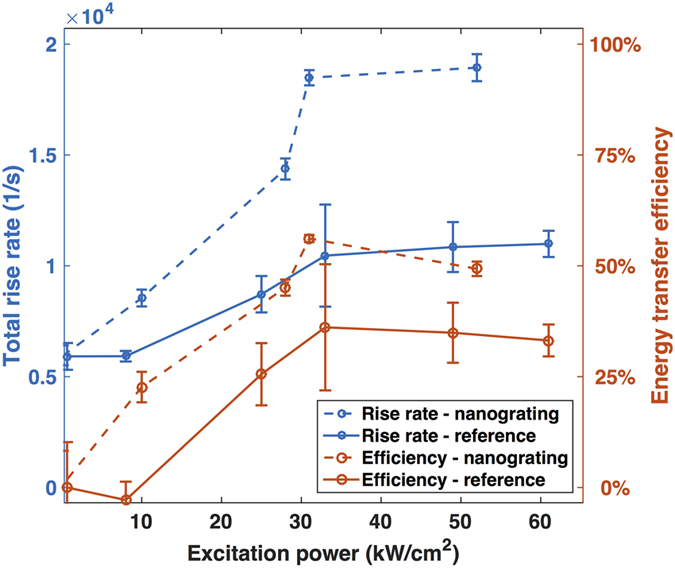
Total rise rate and energy transfer efficiency under different excitation powers. The blue curves referring to the left y-axis correspond to the rise rate of UCNPs on silver nanograting (dash line) and on silver film (solid line) respectively for different excitation power densities. The red curves refereeing to the right y-axis correspond to the internal upconversion efficiency of UCNPs on silver nanograting (dash line) and on silver film (solid line) respectively for different excitation power densities.

The total rise rate 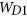 can now be determined by fitting the beginning part of the experimentally measured rise in Fig. 2 with an exponential function. The results are shown in Fig. 4 by blue dash line for nanograting sample, and blue solid line for reference sample. It is clear from equation (11) that the total rise rate of transient NIR PL, 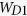, consists of three parts: the intrinsic decay rate, 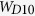, to ground state, the excitation rate 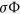, and the ETU rate, 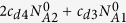. It is straightforward to separate these rates from the total transition rate 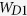. Under weak excitation, the excitation rate should be small. Take the excitation power of 1 kW/cm^2^ for example. The excitation rate is only around 12 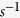, which is more than two orders smaller than the total transition rate 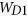 and intrinsic rate 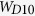. Also, small excitation rate results in small excited state population and consequently small ETU rate. Thus, the total rise is dominated by 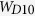. Figure 2 shows that the rise time under weak excitation is close to 170 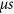 for both nanograting and reference samples. We therefore conclude 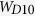 = (170 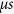)^−1^ = 5.9 × 10^3^ 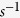. With stronger excitation, the excitation rate increases up to around 3700 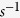 with the strongest excitation condition (61 kW/cm^2^) in our case. Still it contributes less than 20% to the total rise rate 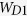. Therefore, the increase of total rise rate under stronger excitation conditions mostly comes from the increased ETU rate, which may be due to the larger population of excited acceptors, 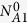 and 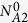, and also the enhanced energy transfer coefficients, 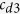 and 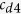. When the excitation is strong enough to start depleting the ground state population of acceptor ions, the population 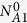 and 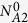 cannot increase further, thus the total rise rate saturates. This is exactly the trend shown in the transient NIR PL in Figs 2 and 3. And this trend has previously been observed in upconverted luminescence system^19^,^37^, irrespective of whether plasmonics is involved. However, with the presence of plasmon resonance, the total rise and decay rates are higher compared to the case without plasmonic nanograting at all excitation conditions. By subtracting the intrinsic rate 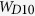 and excitation rate 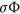 from the total rise rate 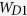, we extract the ETU rate, 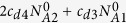. The largest ETU rates are 1.04 × 10^4^ 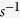 and 3.8 × 10^3^ 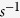 for nanograting and reference samples, respectively. The plasmonic enhancement of ETU rate is around 2.7 fold. This provides direct evidence that the plasmonic field enhances ETU rate in upconverted luminescence. It is noted that the ETU enhancement factor reported here should not be confused with the PL enhancement factors reported earlier. As discussed in detailed in our previous report^12^, the PL enhancement factors depend on the enhancement of absorption and energy transfer coefficient, 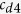. This paper reports the ETU rate, which is a directly measurable quantity defined as the product of energy transfer coefficient and excited state population.

Among the processes governing the population of intermediate energy levels, only the ETU process leads to the desired visible photon emission while the radiative and non-radiative decays both act as loss mechanisms. We define the internal upconversion efficiency as the ratio of ETU rate to total NIR transition rate.


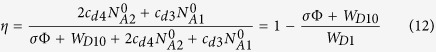


The efficiencies are shown in Fig. 4 by orange dash line for nanograting sample, and orange solid line for reference sample. The internal upconversion efficiency is close to zero under weak excitation, where decay processes dominate. The efficiency becomes higher under stronger excitation until it reaches the saturation value of 36% for UCNPs on silver film, and 56% for UCNPs on silver nanograting. The presence of plasmon resonance enhances the internal upconversion efficiency by 1.6 fold. Since the internal upconversion efficiency is already high under the strong excitation condition even without the enhancement from plasmonics, the maximum possible enhancement is limited to about 3x. Under weak excitation conditions, which correspond to, for example, the solar cell operating condition, much greater enhancement is possible.

The analysis on the decay process can be done similarly. It reveals single exponential decay at the beginning of the NIR decay process, and the decay rate equals to the rise rate obtained from the transient NIR PL rise discussed before. While consistent with the rise analysis, it provides no additional information. Nevertheless, we provide the rate equation analysis of the decay process in the supplementary information for completeness.

### Transient green emission

**Figure 5:**
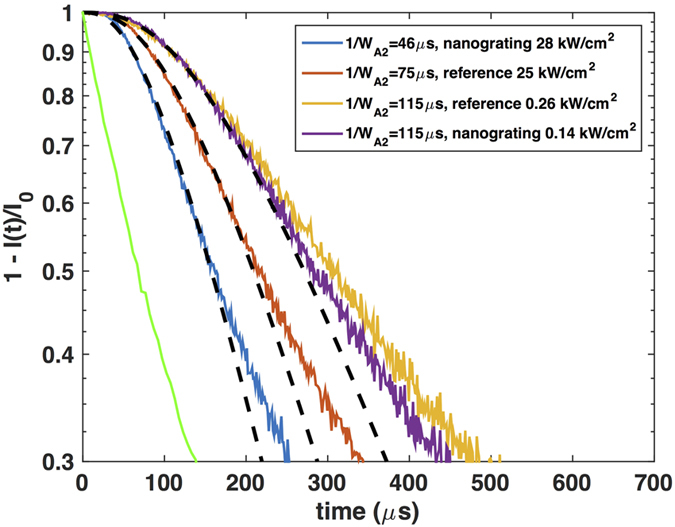
Rise of green emission monitored at 550 nm for UCNPs on silver nanograting sample and on reference sample under excitation of a 980 nm laser with different power densities. The rise curves shown in the figure are normalized to steady state green PL intensities, and subtracted by unity, 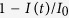. The intrinsic green emission decay measured under a 532 nm green laser excitation is shown by the green line with a decay time of 95 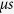. The black dash lines are fitting curves based on the analytical solution (Eq.14).

The transient green PL depends on three processes: ETU to excite the Er^3+^ ions to the emitting levels, radiative decay from the ^4^S_3/2_, ^2^H_11/2_ levels to ground state ^4^I_15/2_ while emitting green photons, and non-radiative decay to intermediate levels and ground state. The total green decay rate, including both the radiative and non-radiative decays, can be directly measured by monitoring the decay of green emission under green laser pulse excitation. We used a 532 nm laser to excite Er^3+^ ions from ground state ^4^I_15/2_ level directly into ^2^H_11/2_ level, and monitored the subsequent green emission from Er^3+^ ions in the ^2^H_11/2_ and ^4^S_3/2_ levels. In this case, the green luminescence exhibits a single exponential decay as shown in Fig. 5 (green line) with a decay time of 95 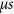.

To investigate the influence of ETU on the transient green emission, we measured the rise of the green emission under 980 nm laser excitation with different excitation conditions as shown in Fig. 5. The experimental details are presented in Methods. Again, the experimentally measured green PL rise is normalized by the steady state PL intensity, and subtracted from unity so that the rise rates may be extracted. For all excitation conditions, the rise begins slowly because of the competition between ETU and decay. At later times, the rise rate becomes similar to the decay rate (Fig. 5, green line). Also, with stronger excitation, the green rise approaches the decay rate, which corresponds to the case of infinitely fast ETU rate. This indicates faster ETU rates under stronger excitation, and is consistent with what we observed in the transient NIR PL. For a similar excitation power density around 25 kW/cm^2^, the rise rate of green emission from nanograting sample is significantly faster than that from the reference sample. It again implies enhanced ETU rate by the plasmon resonance. Under weak excitation, the rise rate of green emission is similar for both samples.

For quantitative study of the green emission dynamics, we perform the rate equation analysis of the transient green emission rise processes. The relevant rate equation for the green emission is equation (5), and we re-write here:


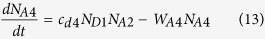


Here, 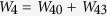 is the total decay rate of green emission. The measured value of W_4_ under the excitation by green laser was (95 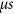)^−1^ = 1.05 × 10^4^ 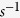 as shown in Fig. 5. Once again, we approximate the intermediate level population of donor and acceptor ions 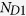 and 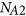 with single exponential functions, 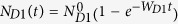 and 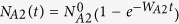. Here, 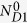 and 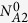 are the corresponding steady state population at 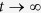. 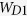 and 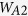 are the rise rates of 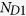 and 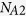 respectively. Substituting the expression of 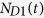 and 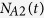 into equation (13), and solving for normalized green emission population 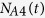 yields,


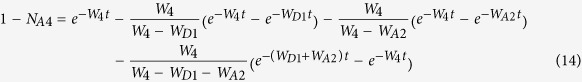


In equation (14), the green decay rate 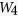 and donor NIR transition rate 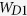 are directly measured from experiments as described previously. By fitting the experimental green emission rise curves in Fig. 5 with equation (14), we obtain acceptor NIR transition rate 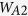 as listed in the Table 1 for different excitation conditions. As we increase the excitation power, the acceptor NIR transition rate 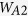 becomes faster as 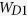 does. But the ratio of the acceptor transition rate 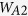 to donor transition rate 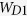 remains the same at 1.5 for all excitation conditions for both samples, irrespective of the use of plasmonic nanostructure. These results indicate the donor and acceptor transition rates are affected in the same way by the excitation power density and plasmon enhancement, which is not unexpected.

In addition to the study of the transient green emission rise process, we also analyzed the decay process. The calculated NIR transition rate of acceptor ions from decay process is the same as the value we obtained from rise process analysis. As this analysis does not yield any new information, they are provided in the SI for completeness.

Finally, we discuss possible temperature effects due to the inadvertent heating that may arise from the absorption of light by metal. As commonly observed in luminescent systems^38^,^39^,^40^,^41^, the photoluminescence is temperature dependent. Fortunately, the UCNP temperature can be accurately determined by the ratio of two green emission bands (I_S_: ^4^S_3/2_→^4^I_15/2_, I_H_: ^4^H_11/2_→^4^I_15/2_). Due to the close proximity of their energy levels, the two green emission bands arising from the ^4^S_3/2_ and ^4^H_11/2_ levels follow the Boltzmann population distribution^42^,^43^,^44^


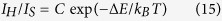


Here, 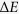 = 600 cm^−1^ is the energy gap between level ^4^S_3/2_ and ^4^H_11/2_, and C is constant.

The ratio, I_H_/I_S_, is shown in SI Fig. S8 for all excitation conditions used in this paper, 1 kW/cm^2^ ~ 61 kW/cm^2^. The weakest excitation power density, 1 kW/cm^2^, is deep in the weak excitation regime and it is thus reasonable to assume the temperature of the sample under the weakest excitation is equal to the room temperature^13^, 300 K. By converting the intensity ratios into the temperature, we obtain the sample temperatures under all other excitation conditions shown in SI Fig. S9. The temperature of the reference sample remains almost the same for the entire excitation power density range while a small increase was observed from the nanograting sample. However, the largest temperature increase in our case was still less than 16 K. Therefore, we believe that the thermal effects under high excitation power density is not important in our study.

## Discussion

The luminescence upconversion system we studied here is the most efficient mechanism of ETU where two sensitizer ions (donors) transfer energy to a single activator ion (acceptor) successively to achieve frequency upconversion. The ETU is composed of three distinct physical processes: absorption by sensitizers, successive energy transfer from two sensitizers to an activator, and emission by the activator. Surface plasmon can affect all three processes. When the surface plasmon resonance is tuned to the absorption wavelength, it can enhance both the absorption and energy transfer processes. Absorption enhancement arises from the local field enhancement, which increases the local intensity and consequently absorption. Surface plasmon can also enhance energy transfer process. The exact mechanism of plasmon enhancement of energy transfer process has been disputed. An earlier work attributed it to enhanced local density of states (LDOS)^45^. But more recent works showed LDOS does not influence energy transfer rate^46^,^47^. Nevertheless, many theoretical and experimental studies showed plasmon resonance does influence energy transfer rate^12^,^13^,^48^,^49^. This work represents the first experimental demonstration and quantification of plasmon enhanced energy transfer rate in UCNPs. Finally, when plasmon resonance is tuned to the emission wavelength, emission rate can be enhanced *via* the Purcell effect. We present below a more detailed discussion on each process.

The absorption depends on the local light intensity, absorption cross-section of the active ions and the thickness of the upconversion material. Lanthanide ions generally exhibit small absorption cross-section, due in large part to the forbidden nature of the f-f transitions. Take, for example, Na_2_Y_3_F_11_:18%Yb^3+^,2%Er^3+^ crystal, which is one of the most efficient upconversion materials, an absorption coefficient of 6 cm^−1^ has been reported^10^. For 1 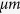 thickness sample, only 0.06% of the incident light gets absorbed. An attractive method to improve the absorption efficiency is to enhance the local field by tuning the plasmon resonance to the absorption frequency^12^,^13^,^50^. The plasmon mode can generate hot spot near the metal-dielectric interface where upconversion material may be placed. The higher local field naturally leads to more absorption. The absorption enhancement can be directly measured experimentally as we reported earlier^12^. Due to the momentum mismatch, the coupling of excitation laser to plasmon mode is negligible for flat film reference sample used in this report. Periodic structure is an efficient method to couple excitation into plasmon resonance. A simple silver nanograting structure used in this report was shown to enhance the absorption by 3 fold. Higher absorption enhancement can be achieved with more sophisticated plasmonic structure design.

For the emission processes, the efficiency is determined by the ratio of radiative decay rate to all other non-radiative decay rates. Ideally, we want to maximize the radiative decay rate and suppress the non-radiative decay rates as much as possible. One major part of the non-radiative decay mechanism is the quenching by defects. By using, for example, core-shell structure^32^,^51^, the quenching by surface defects can be suppressed. It is also possible to enhance the emission rate by tuning plasmon resonance to the emission frequency^16^. The plasmon mode enhances the local density of photon modes, leading to faster radiative decay rate and higher emission efficiency. The enhancement of the emission rate can be directly visualized from the measurement of PL decay following a pulsed excitation, if sufficiently large enhancement is achieved so that the radiative decay rate exceeds non-radiative rates. In this report, the plasmon resonance is far away from the emission frequencies so that no plasmon effect on emission process is expected.

While the understanding of absorption and emission efficiency is relatively straightforward, the energy transfer efficiency has remained difficult to quantify. We have analytically calculated the plasmon effect on energy transfer rate for flat silver film^12^,^19^, which shows the ability of substantial enhancement near the plasmon resonance of silver while no effect in other frequencies, e.g. excitation at 980 nm. For more complicated structure such as nanograting, no analytical solution of energy transfer rate is available so that experimental measurement is in strong desire. In this report, we present a detailed rate equation based analysis of the transient NIR PL, and present a method to experimentally determine the ETU rate and internal upconversion efficiency for various excitation conditions. The internal upconversion efficiency specifies the fraction of rate at which the acceptor ions are excited from the intermediate level to the emitting level. It provides a quantitative metric to describe the competition between the ETU rate and other loss mechanisms, including the radiative decay of the excited donor and acceptor ions, cross-relaxation between acceptor ions, and the non-radiative energy transfer to metal and defects. This study reveals that the ETU rate increases with increasing excitation intensity until it reaches saturation, where the highest internal upconversion efficiency is achieved. The highest efficiency is 56% with the enhancement by the plasmonic nanograting, while it remains at 36% for UCNPs in the reference sample. The internal upconversion efficiency and ETU rate are enhanced by 1.6 fold and 2.7 fold, respectively, by the plasmonic nanograting. The 36% internal upconversion efficiency exhibited by the reference sample indicates that the maximum achievable enhancement is about three-fold when operating under strong excitation conditions. Under weak excitation conditions, much greater enhancement should be possible. To our knowledge, this is the first experimental measurement of plasmon enhanced energy transfer upconversion rate and internal upconversion efficiency. The clear understanding and quantification of the plasmon enhancement of energy transfer process paves the way for more advanced design of plasmonic enhanced upconversion luminescence materials.

## Methods

The details of sample preparation, including UCNPs synthesis, UCNPs surface treatment, silver nanograting fabrication, LBL deposition of UCNPs, and reflectance spectral characterization can be found in our previous report^12^.

### Steady-state PL spectroscopy

We excite the UCNPs samples with a 980 nm laser diode (OEM laser) normally incident on the sample surface. The polarization is in the direction orthogonal to silver nanograting lines to excite the surface plasmon modes. For visible PL measurement, the excitation light reflected off the sample surface is rejected by a dichroic mirror (Thorlabs DMSP 805) while the visible PL signal is transmitted through the dichroic mirror and collimated by a convex lens with focal length of 5 cm. The collimated PL signal is then focused by another convex lens into the entrance slit of spectrometer (Acton SpectraPro 300i). A liquid nitrogen cooled Si CCD (Roper Scientific) is equipped with the spectrometer to detect and record the PL spectrum. In order to protect the detector from any stray laser light, short-pass edge filters (Scott KG5) are placed right before the spectrometer entrance slit. Same setup is used for NIR PL measurement with minor changes. The excitation laser is first cleaned up by a band-pass filter (Semrock LL01-980) with 3.7 nm bandwidth centered at 980 nm. Then, we use NIR beam splitter cube, instead of dichroic mirror, to reflect the excitation laser to samples. The emitted PL signal from the sample passes through the beam splitter cube, and get collected to the spectrometer. In order to protect the NIR detector (Andor iDus InGaAs), we put one long-pass filter (Semrock BLP01-980R) before the monochromator entrance slit to attenuate the laser light and pass only the NIR emission from UCNPs.

### Time-resolved PL spectroscopy

In the time-resolved PL spectroscopy, a square pulse train of 980 nm light from laser diode (Thorlabs L980P200) is used to excite UCNP samples at normal incidence. The laser pulse duration and duty cycle are controlled by a function generator (Wavetak model 166) connected to the laser diode current driver (Thorlabs TCLDM9). For the transient NIR PL reported in this paper, we used a square wave with 20 ms period and 50% duty cycle. A band-pass filter (Semrock LL01-980) with 3.7 nm bandwidth centered at 980 nm is placed in front of the laser to filter out side band emissions. Then, the cleaned laser beam is expanded by a 5x achromatic Galilean beam expander to the diameter around 1 cm before it is focused tightly onto the sample surface. The excitation laser beam is directed onto the sample at normal incidence using a NIR beam splitter cube. For the plasmonic nanograting sample, we align the laser polarization orthogonal to the nanograting lines. The emission from the UCNPs is collimated and re-focused to a monochromator (Sciencetech 9057F) by two convex lenses. For transient NIR PL detection, we place one long-pass filter (Semrock BLP01-980R) before the monochromator entrance slit to attenuate the laser light and pass only the NIR emission from UCNPs. The monochromator is set at 1002 nm and the transient PL signal is recorded by a NIR photomultiplier tube (Hamamatsu H10330B-75), which is connected to a photon counter (Stanford Research Systems SR430). The photon counter input is synchronized with the laser pulse by the function generator, and the transient NIR PL is stored in the internal memory of photon counter. The same setup is also used for the transient green PL detection with the same 980 nm laser pulse excitation except that we replace the long-pass filter (Semrock BLP01-980R) by short-pass edge filters (Scott KG5), and deliver 550 nm light to visible PMT (Hamamatsu H11461P-11). The 980 nm laser excitation power is adjusted by the function generator output to laser diode current drive. We also measured the transient green PL under green laser (Thorlabs, DJ532-40) excitation at 532 nm using the same setup. The side band of excitation green laser is filtered by band-pass filter (Semrock FF01-524/24). Before the monochromator entrance, the excitation green laser is attenuated by long-pass filter (BLP01-532R-25). For the green laser pulses, the square waveform has a period of 40 ms with 50% duty cycle.

## Additional Information

**How to cite this article**: Lu, D. *et al.* Experimental demonstration of plasmon enhanced energy transfer rate in NaYF_4_:Yb^3+^,Er^3+^ upconversion nanoparticles. *Sci. Rep.* **6**, 18894; doi: 10.1038/srep18894 (2016).

## Supplementary Material

Supplementary Information

## Figures and Tables

**Table 1:** NIR transition rate of donor and acceptor under different excitation power.

	Nanograting, 0.14 kW/cm^2^	Reference, 0.26 kW/cm^2^	Nanograting, 28 kW/cm^2^	Reference, 25 kW/cm^2^
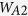	8.70 × 10^3^ s^−1^	8.70 × 10^3^ s^−1^	2.17 × 10^4^ s^−1^	1.33 × 10^4^ s^−1^
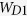	5.88 × 10^3^ s^−1^	5.88 × 10^3^ s^−1^	1.43 × 10^4^ s^−1^	8.70 × 10^3^ s^−1^
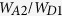	1.48	1.48	1.52	1.53

The sample information and excitation conditions are listed in first row. The second row lists the NIR transition rate 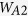 of acceptor ions fitted from the transient green emission rise curves. The third row lists the NIR transition rate 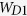 of donor ions obtained from previous transient NIR emission analysis. The last row lists the ratio of these two rates.
