# Supplementary material for: Experimental demonstration of plasmon enhanced energy transfer rate in NaYF4:Yb3+,Er3+ upconversion nanoparticles
Source: Sci Rep. 2016 Jan 7;6:18894. doi: 10.1038/srep18894 (PMC4703967; doi:10.1038/srep18894)
Supplement: Supplementary Information [file srep18894-s1.pdf]

Supplementary information

Experimental demonstration of plasmon enhanced energy transfer rate in $NaYF_4:Yb^{3+},Er^{3+}$ upconversion nanoparticles

Dawei Lu,[1] Sungmo Ahn,[1] Chenchen Mao,[1] Suehyun K. Cho[1] and Wounjhang Park,[1,2,*]

[1]Department of Electrical, Computer and Energy Engineering

[2]Materials Science and Engineering Program

University of Colorado, Boulder, CO 80309-0425, U.S.A.

1. Rate equation analysis of the NIR PL decay.

The NIR PL decay follows the same equation as the rise except the pumping term:

$$\frac{dN_{D1}}{dt} = -W_{D10}N_{D1} - c_{d3}N_{D1}N_{A1} - 2c_{d4}N_{D1}N_{A2} \tag{1}$$

During the decay process, the population of N_{A2} and N_{A1} can be approximated by single exponential as $N_{A2}(t) = N_{A2}^0 e^{-W_{A2}t}$ and $N_{A1}(t) = N_{A1}^0 e^{-W_{A1}t}$ respectively. Here, N_{A2}^0 and N_{A1}^0 are the steady state population of intermediate energy levels A_2 and A_1 at t = 0, and W_{A2} and W_{A1} are the corresponding decay rates. Directly solving the equation (1), we obtained the normalized population of N_{D1}:

$$N_{D1}(t) = \exp\left[-W_{D10}t - \frac{2c_{d4}N_{A2}^0}{W_{A2}}\left(1-e^{-W_{A2}t}\right) - \frac{c_{d3}N_{A1}^0}{W_{A1}}\left(1-e^{-W_{A1}t}\right)\right] \tag{2}$$

For $t \ll \min\{1/W_{A2}, 1/W_{A1}\}$, the normalized population of N_{D1} can be approximated by

$$N_{D1}(t) = \exp\left[-\left(W_{D10} + 2c_{d4}N_{A2}^0 + c_{d3}N_{A1}^0\right)t\right] \tag{3}$$

The total decay rate $W_{D1,decay}$ of NIR PL is then given as,

$$W_{D1,decay} = W_{D10} + 2c_{d4}N_{A2}^0 + c_{d3}N_{A1}^0 \tag{4}$$

This is exactly the same as the rise rate expressed in equation (11) in the manuscript, except for the excitation term.

2. Discussion on the exponential fit of the transient NIR PL

Both the NIR decay and rise are essentially non-exponential but can be approximated by single exponential functions at the very beginning. Furthermore, the NIR decay deviates from single exponential function quicker than the NIR rise does as we mentioned in the manuscript. Here, we provide a more in-depth discussion on the single exponential approximation used to fit the initial part of the transient PL in this paper.

The normalized NIR rise curve can be expressed as $I_{rise}(t) = 1 - e^{-W_{rise}t}$ where the rise rate W_{rise} is given by equation (10) in the manuscript (denoted as W_{D1}). We reproduce it here for readability:

$$W_{D1} = W_{D10} + 2c_{d4}N_{A2}^{0}\left[1 - e^{-W_{A2}t} + e^{(W_{D1}-W_{A2})t}\right] + c_{d3}N_{A1}^{0}\left[1 - e^{-W_{A1}t} + e^{(W_{D1}-W_{A1})t}\right] \quad (5)$$

On the other hand, the normalized NIR PL decay has the expression given in equation (2) above. The overall decay rate may be written as,

$$W_{decay} = W_{D10} + 2c_{d4}N_{A2}^{0}\frac{1 - e^{-W_{A2}t}}{W_{A2}t} + c_{d3}N_{A1}^{0}\frac{1 - e^{-W_{A1}t}}{W_{A1}t} \quad (6)$$

By comparing the rise rate and the decay rate, we find that the time dependent coefficient before the energy transfer terms, $2c_{d4}N_{A2}^{0}$ and $c_{d3}N_{A1}^{0}$, are different. Take the term $2c_{d4}N_{A2}^{0}$, for example. The coefficients are $1 - e^{-W_{A2}t} + e^{(W_{D1}-W_{A2})t}$ and $\frac{1 - e^{-W_{A2}t}}{W_{A2}t}$ for the rise and decay rate, respectively. For $t \to 0$, retaining only up to the first order terms yields single exponential functions. However, the higher order terms, which become more

important as t is increased, behave quite differently. The deviations from the single exponential functions are shown in Figure S1 for the nanograting sample with an excitation power of 52 kW/cm^2. Both the rise and decay stay close to single exponential up to a few tens of microseconds. For t > 50 μs, the decay continues to deviate from single exponential while the rise remains reasonably close to single exponential. This explains the faster deviation from single exponential we observed in NIR decay PL, and also provides the justification for our choice of rise curves to conduct the detailed analysis.

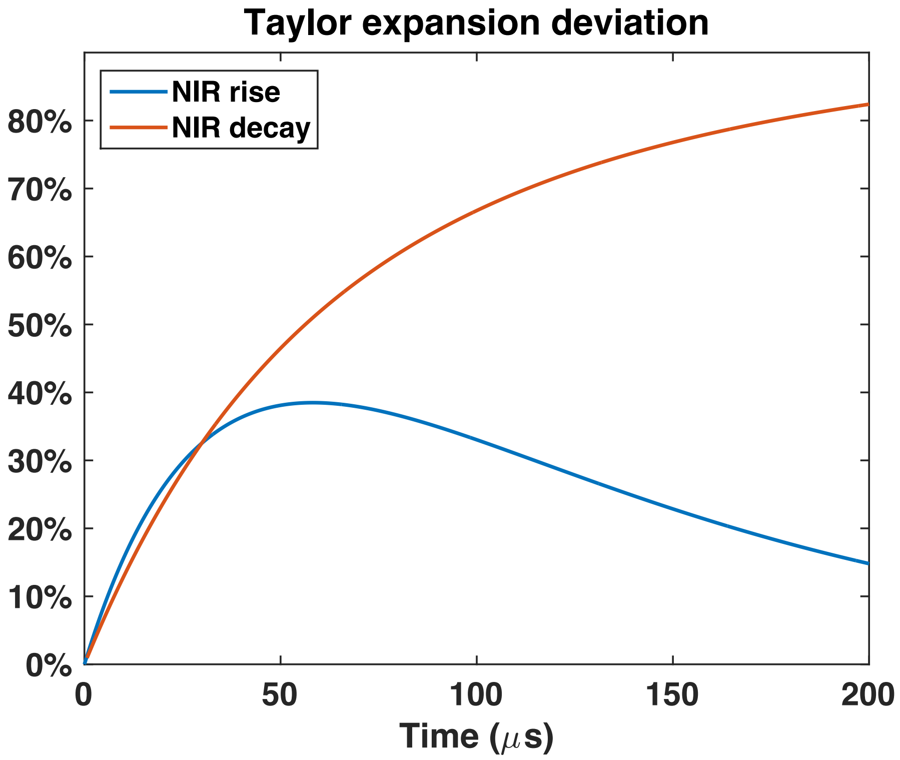

Figure S1. The deviation of 1st order Taylor expansion from actual value for coefficient in energy transfer term $2c_{d4}N_{A2}^{0}$ in NIR rise and decay analytical solutions for nanograting sample under 52 kW/cm2 excitation.

3. Analysis of green PL decay

The decay curves of green emission under 980 nm laser excitation are shown in Figure S2 for the nanograting and reference samples. Again, we observe slow decay at the beginning as a result of competition between upconversion and decay. Later, all decay curves follow the intrinsic decay as shown in green line in Figure S2.

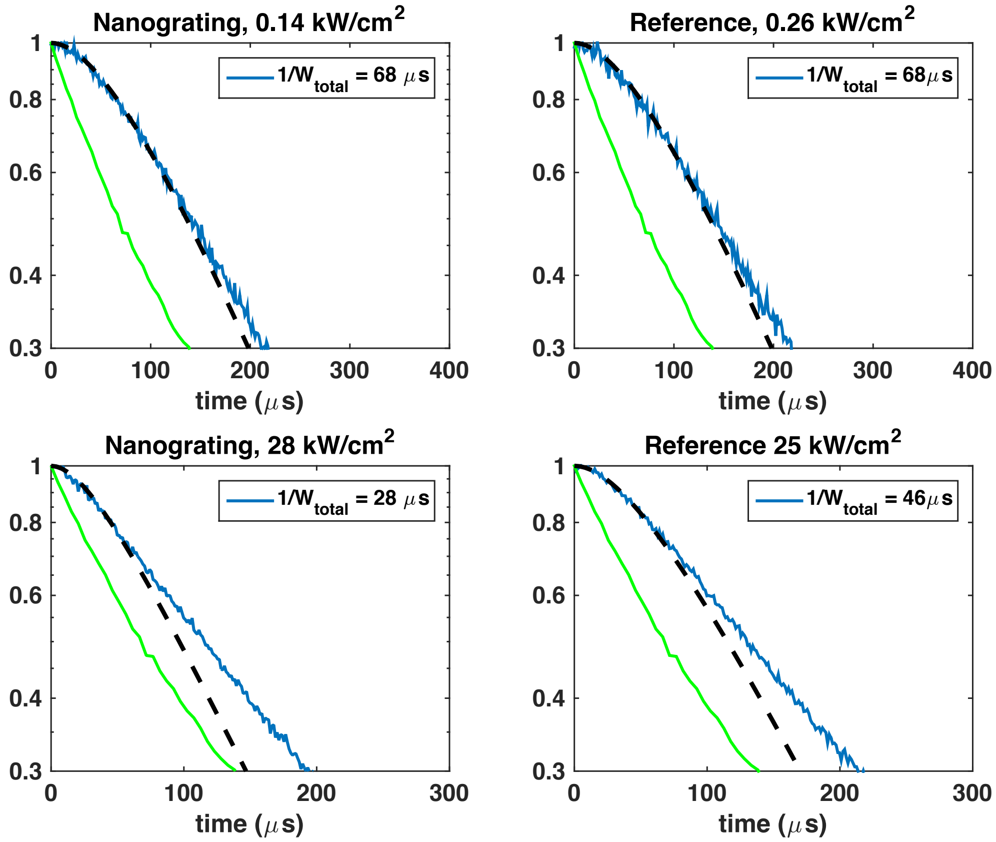

Figure S2. Green emission decay (blue lines) of UCNPs on nanograting (nanograting sample) and on silver film (reference sample) under weak and strong excitation power densities. The green emission decay of UCNPs upon green laser excitation is a single exponential decay with a decay time of 95 μs (green line). The black dash lines are the fittings using equation (9). All experimental PL decay curves are normalized with the steady state PL intensities, and plotted in logarithmic scale.

The equation (13) in manuscript applies for green emission decay process as well. We re-write it here:

$$\frac{dN_{A4}}{dt} = c_{d4} N_{D1} N_{A2} - W_{A4} N_{A4} \tag{7}$$

The description and the values of the coefficients can be found in manuscript. In the decay process, the intermediate energy level population, N_{D1} and N_{A2}, can be approximated by single exponential decay: $N_{D1}(t) = N_{D1}^0 e^{-W_{D1}t}$ and $N_{A2}(t) = N_{A2}^0 e^{-W_{A2}t}$ where N_{D1}^0 and N_{A2}^0 are the steady state population of *D1* and *A2* levels at $t = 0$, and W_{D1} and W_{A2} are the decay rate of N_{D1} and N_{A2} respectively. The ETU term can then be written as,

$$c_{d4} N_{D1} N_{A2} = c_{d4} N_{D1}^0 N_{A2}^0 e^{-(W_{D1}+W_{A2})t} \tag{8}$$

Substituting this expression for $c_{d4} N_{D1} N_{A2}$ into equation (7), and solving for the normalized decay expression of green emission yields,

$$N_{A4}(t) = \frac{W_{A4}}{W_{A4} - W_{total}} \left[e^{-W_{total}t} - \frac{W_{total}}{W_{A4}} e^{-W_{A4}t} \right] \tag{9}$$

where W_{total} is the sum of decay rates W_{D1} and W_{A2}.

The experimentally measured green decay was fitted by equation (9) to extract the total decay rate W_{total}. Since we had measured the NIR emission decay of donor ions W_{D1}, we can subtract it from W_{total} to obtain acceptor ion NIR decay rate W_{A2}. The results are listed in Table S1. As we can see, the NIR decay rates of acceptor ions equal to its rise rates presented in the manuscript. And once again the ratio of NIR decay rates of acceptor ions to that of donor ions stay constant at 1.5 irrespective of the excitation conditions.

	Nanograting, 0.14 kW/cm^2	Reference, 0.26 kW/cm^2	Nanograting, 28 kW/cm^2	Reference, 25 kW/cm^2
W_{total}	1.47 x 10^4 s^{-1}	1.47 x 10^4 s^{-1}	3.57 x 10^4 s^{-1}	2.17 x 10^4 s^{-1}
W_{D1}	5.88 x 10^3 s^{-1}	5.88 x 10^3 s^{-1}	1.41 x 10^4 s^{-1}	8.93 x 10^3 s^{-1}
$W_{A2}=W_{total}-W_{D1}$	8.85 x 10^3 s^{-1}	8.85 x 10^3 s^{-1}	2.16 x 10^4 s^{-1}	1.28 x 10^4 s^{-1}
W_{A2}/W_{D1}	1.51	1.51	1.53	1.43

Table S1. The sample information and excitation conditions are listed in first row. The total NIR decay rates obtained from the green emission decay at different excitation power densities are listed on the second row. The third row shows the NIR decay rate of donor ions from NIR PL analysis presented in manuscript. The acceptor ions NIR decay rates W_{A2} are listed on fourth row. The last row shows the ratio of acceptor NIR decay rates to donor NIR decay rates.

4. UCNP synthesis and surface modification

UCNPs were synthesized using a modified co-precipitation method[1]. 0.3 g of YCl_3, 0.1 g of $YbCl_3$, and 0.01 g of $ErCl_3$ were dissolved into 36 mL of octadecene and 6 mL of oleic acid by vigorous stirring and heating. Then a mixture of 0.2 g of NaOH, and 0.296 g of NH_4F in 20 mL of methanol was added and vigorously stirred for 30 min at 60 ºC. After 30 min, the mixture was heated up to 100 ºC for degassing. Then, the mixture was heated to 320 ºC for an hour under argon atmosphere. Once the final product has cooled down, the mixture was washed in water and ethanol *via* centrifugation and redispersion. After final step of washing, UCNPs were stored in toluene.

As shown in the transmission electron micrographs (TEM) in Fig. S3, the nanoparticles were regular hexagonal platelets, indicating the formation of β-phase

$NaYF_4$ nanocrystals. The mean lateral size was 32 nm. The Yb^{3+} and Er^{3+} doping densities were 18% and 2%, respectively. We also show the high-resolution transmission electron microscopy (HRTEM) image (Hitachi HF2000, Japan) and XRD pattern of the as-synthesized β-$NaYF_4$ in Fig. S4 from previous report[2].The UCNPs were of good optical quality and exhibited strong upconverted luminescence under the excitation at 980 nm. The as-synthesized UCNPs are covered with oleic acid and thus not water-soluble. To make them water-soluble and also to make the nanoparticle surface negatively charged, we coated the nanoparticles with poly(maleic anhydride-*alt*-1-octadecene) (PMAO). The inset of Fig. S3 shows thin and uniform coating of PMAO on UCNPs. The mean coating thickness was 2 nm. The PMAO coated UCNPs can then be deposited uniformly in the layer-by-layer (LBL) fashion with precisely controlled thickness.

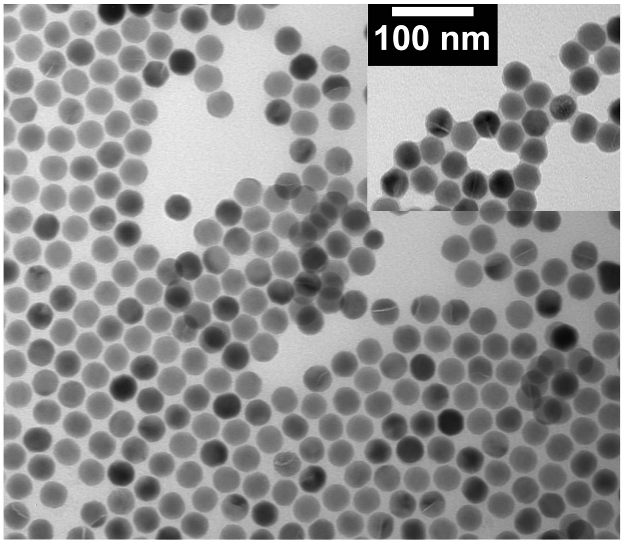

Figure S3. TEM of as-synthesized β-phase $NaYF_4$:Yb^{3+},Er^{3+} UCNPs. The mean lateral size was 32 nm. The inset shows the TEM of PMAO-coated UCNPs showing uniform 2 nm thick coating. The scale bar applies to both TEM images.[6]

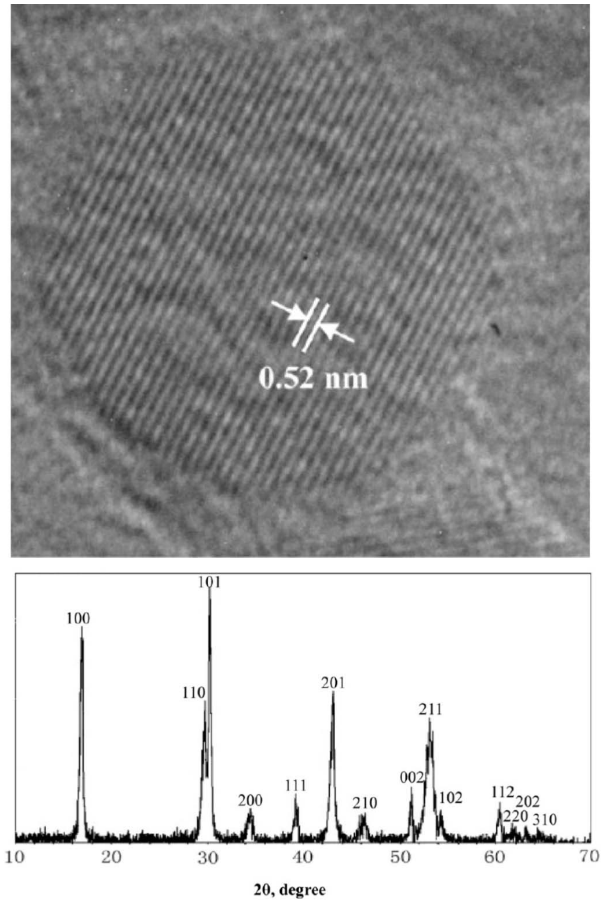

Figure S4. High-resolution TEM and x-ray diffraction of β-$NaYF_4$.[2]

5. Layer-by-Layer Deposition of UCNPs

The layer-by-layer (LBL) deposition process driven by the electrostatic interaction was carried out by using polyelectrolytes as intermediaries.[3-5] Si_3N_4 coated silver nanogratings were washed in acetone overnight and dried in vacuum oven for 3 hours. The cleaned nanogratings were then immersed in 1% (v/v) (3-aminopropyl)triethyoxysilane (APTS) in toluene for 3 hours. Then, the nanogratings were washed with toluene and ethanol and dried in vacuum oven overnight. The APTS coated chips were then stamped with a silicone glue well (diameter ~4 mm) to define the area of nanoparticle deposition. After

drying silicone glue well for 20 min, 6 μL of surface modified UCNP solution was drop-cast and left to react for 20 min. A gentle washing to get rid of unbound nanoparticles was then performed. Then, 6 μL of 2 mg/mL of poly(allylamine hydrochloride) (PAH) solution was drop-cast in the well, reacted for 20 min and gently washed. These procedures were repeated until three monolayers of UCNPs were obtained. Successful deposition of three monolayers in the layer-by-layer fashion was confirmed by the atomic force microscopy performed at each layer. The final thickness of three monolayers was measured to be 90 nm by the atomic force microscopy.

We conducted a series of atomic force microscopy scans to determine the thickness of one, three and five monolayers of UCNP samples. As shown in Fig. S5, the thickness increased linearly with the number of layers. The three-layer sample, which was used in this paper, had a thickness of 90 ± 2 nm.

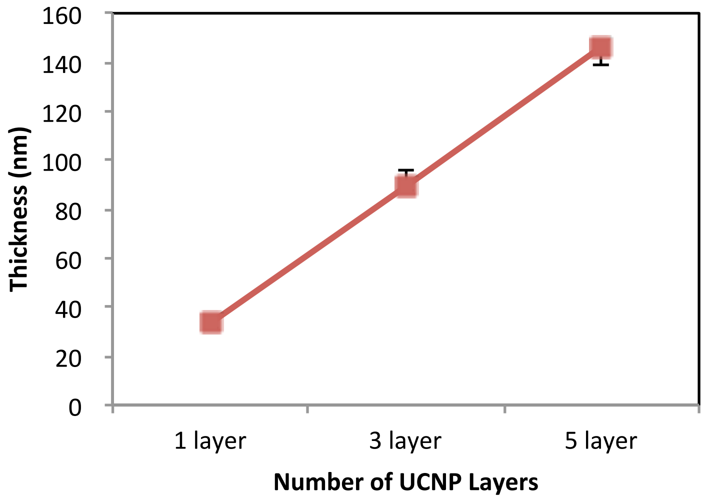

Figure S5. Atomic force microscopy measurements of thicknesses of 1, 3 and 5 layer UCNP films deposited by the LBL method.[6]

6. Nanograting Fabrication

To fabricate the silver nanograting structure, we used the nanoimprint lithography. A 200 nm thick Ag film was deposited on a silicon substrate coated with a 20 nm thick Cr adhesion layer. Another 10 nm thick Au layer was deposited on top to prevent any oxidation of silver during the subsequent oxygen reactive ion etching (RIE) process. Then, a 175 nm thick poly(methylmethacrylate) (PMMA) layer was spin-coated and baked on a hot plate. A silicon mold with a grating structure (LightSmyth Technologies) was used to imprint the PMMA layer and the oxygen RIE was carried out to remove any residual PMMA in the trenches. By evaporating a 20 nm thick Ag film on top of the imprinted PMMA layer and lifting off in acetone, a high-quality silver grating structure was obtained over a large area (0.64 mm^2). The fabricated nanograting has 20 nm thick and 410 nm wide silver lines with a period of 830 nm, as shown in the SEM image in Fig. S6. Finally, a thin Si_3N_4 layer was deposited on the silver gratings by plasma-enhanced chemical vapor deposition to alleviate luminescence quenching. We also performed reflectance spectrum measurement of the as-fabricated nanograting and complete structure coated with Si_3N_4 and UCNPs in Fig. S7. The results matches simulated reflectance spectra well.

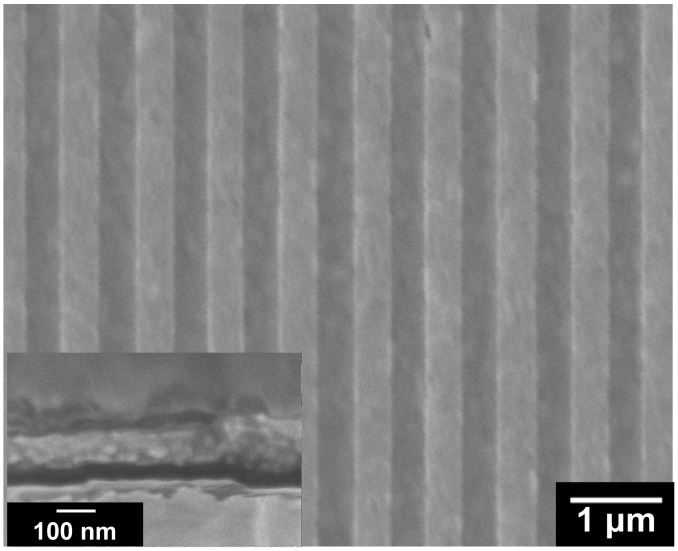

Figure S6. SEM of silver nanograting with period of 830 nm and line width of 410 nm. The inset shows the cross-sectional SEM of grating-Si_3N_4-UCNPs. The black layer in the middle is 30 nm Si_3N_4 coating sitting on top of the nanograting. Above the Si_3N_4 layer are the three monolayers of UCNPs. The thickness was consistent with the atomic force microscopy measurements. To acquire good quality images, a thick gold overlayer was deposited on top of UCNPs for this sample.[6]

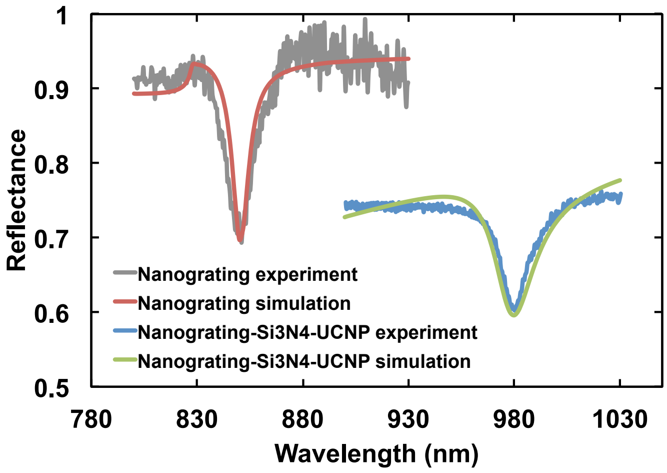

Figure S7. Experimental reflectance spectra of as-fabricated nanograting (gray) and nanograting-Si_3N_4-UCNP structure (blue). The simulated reflectance spectra for the two structures are also plotted with red and green lines.[6]

7. Temperature of UCNPs

As described in the manuscript, the UCNP temperature can be extracted from the ratio of two green emission bands (I_S: $^4S_{3/2} \rightarrow {}^4I_{15/2}$, I_H: $^4H_{11/2} \rightarrow {}^4I_{15/2}$)

$$I_H / I_S = C \exp\left(-\Delta E / k_B T\right)$$

Here, $\Delta E = 600\ cm^{-1}$ is the energy gap between level $^4S_{3/2}$ and $^4H_{11/2}$, and C is constant.

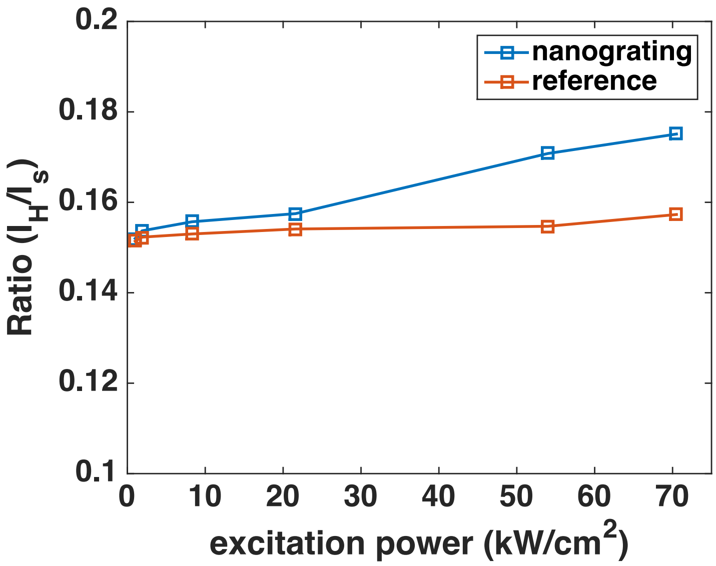

Figure S8. Intensity ratio of two green emission band under different excitation power densities for nanograting sample and reference samples.

The ratio, I_H/I_S, is shown in Fig. S8 for all excitation conditions used in this paper, 1 kW/cm^2 ~ 61 kW/cm^2. Assuming the temperature of the sample under the weakest excitation (1 kW/cm^2) is equal to the room temperature, 300K, we obtain the sample temperatures under all other excitation conditions as shown in Fig. S9.

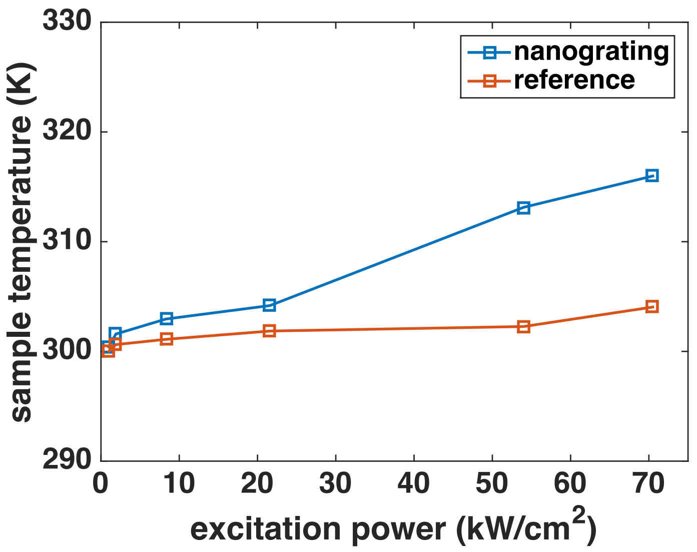

Figure S9. Sample temperature under various excitation conditions.

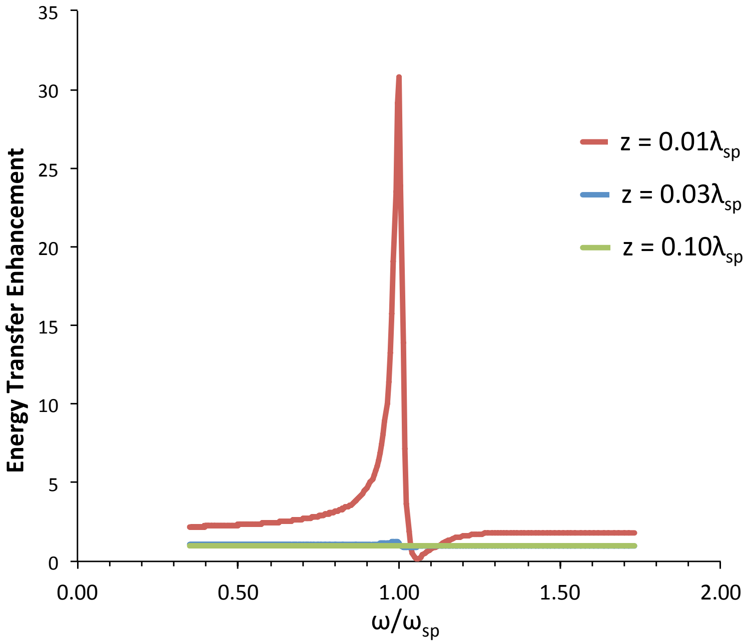

Figure S10. Energy transfer rate enhancement factor calculated for a donor-acceptor pair with a pair separation of 3.4 nm placed at various distances, z, from the silver surface. ω_{sp} and λ_{sp} represent the surface plasmon frequency and wavelength, respectively[6]. For the present case, λ_{sp} = 340 nm.

References

1. Li, Z. & Zhang, Y. An efficient and user-friendly method for the synthesis of hexagonal-phase $NaYF_4$:Yb, Er/Tm nanocrystals with controllable shape and upconversion fluorescence. *Nanotechnology* **19,** 345606 (2008).
2. Li, Z., Park, W., Zorzetto, G., Lemaire, J. S. & Summers, C. J. Synthesis Protocols for δ-Doped $NaYF_4$:Yb,Er. *Chem. Mater.* **26,** 1770–1778 (2014).
3. Ariga, K., Hill, J. P. & Ji, Q. Layer-by-layer assembly as a versatile bottom-up nanofabrication technique for exploratory research and realistic application. *Phys. Chem. Chem. Phys.* **9,** 2319 (2007).
4. Shenhar, R., Norsten, T. B. & Rotello, V. M. Polymer-mediated nanoparticle assembly: structural control and applications. *Adv. Mater.* **17,** 657–669 (2005).
5. Park, W. *et al.* Controlled self-assembly of gold nanoparticles mediated by novel organic molecular cages. *Opt. Mater. Express* **3,** 205–215 (2013).
6. Lu, D. *et al.* Plasmon enhancement mechanism for the upconversion processes in $NaYF_4$:Yb^{3+},Er^{3+} nanoparticles: Maxwell versus Förster. *ACS Nano* **8,** 7780–7792 (2014).
